# The Apoptotic and Antiproliferative Effects of Capsaicin in the Developmental Stages of Oral Squamous Cell Carcinoma Induced in Hamsters

**DOI:** 10.7759/cureus.26073

**Published:** 2022-06-19

**Authors:** Amer Takkem, Safa Zakaraia, Ali Silan, Mohammad Alghazawi, Wisaam Sahyouni, Ahmad AL-Manadili

**Affiliations:** 1 Department of Oral Histology and Pathology, Damascus University, Damascus, SYR; 2 Department of Oral and Maxillofacial Surgery, Damascus University, Damascus, SYR; 3 Department of Orthodontics, Damascus University, Damascus, SYR

**Keywords:** apoptosis, proliferation, capsaicin, oral cancer, hamster

## Abstract

Background and aim

Several epidemiological and experimental studies have approve that the vegetarian diet has an anticancer effect. Capsaicin is the active botanical ingredient found in red chili peppers. While the data strongly argue for the significant anticancer benefits of capsaicin, nevertheless, much information is required to shed light on the anticancer molecular mechanisms to improve knowledge and suggest potential therapeutic mechanisms for the use of capsaicin against cancer. This study aimed to investigate the effect of capsaicin on the rate of cell division and apoptosis in the development of oral squamous cell carcinoma induced in the buccal pouch of hamsters.

Materials and methods

The sample consisted of two groups; the first group consisted of 20 hamsters with the application of carcinogenic 7,12-dimethylbenz(a)anthracene (DMBA) in the buccal pouch (the control group) and the second group (the study group) also consisted of 20 hamsters with the application of DMBA in alternatively with capsaicin. Tissue biopsies were taken from experimental animals after sacrificing. The samples were immunostained for the detection of Ki-67 and Bcl-2 proteins.

Results

Immunohistochemical staining by monoclonal antibody to Ki-67 and Bcl-2 in the study group showed lower expression at all stages of oral cancer development compared with their expression in the control group. After performing the one-way (ANOVA) test, we found statistically significant differences by comparing the expression degree of Ki-67 and Bcl-2 proteins in both study groups, where the p-value was less than 0.05.

Conclusion

We conclude from the data of our study that capsaicin has an anti-cancer role in oral squamous cell carcinoma if applied in the digestive tract of experimental animals by inhibiting the proliferation of cancer cells and activating apoptosis in them.

## Introduction

Several epidemiological studies have shown that individuals who follow a fruit and vegetable diet have a significantly lower risk of developing cancer than others [1]. Strong evidence from literature confirms the importance of phytochemicals to suppress various stages of cancer [2]. Phytochemicals could be found in fruits, vegetables, whole grains, spices, and tea. They have shown diverse inhibitory effects against carcinogenesis, growth, progression, and colonization of malignancy [3]. Capsaicin (trans-8-methyl-n-vanillyl-6-nonenamide) is a homovanillic acid derivative and is extracted primarily from red chili peppers and has been used medicinally for centuries and extensively as a pain reliever and antiinflammatory, antioxidant, and antiobesity. Moreover, it has been proven recently its anticancer properties and has effective results in treating a variety of cancers [4]. Capsaicin is the main pungent component of hot peppers, soluble in alcohol and fat, with a molecular weight of 305.40 g/mol [5]. Capsaicin is easily absorbed in the gastrointestinal tract, passes through cell membranes, and reaches a peak plasma concentration after 45 minutes [6]. Although the effect on tumor growth is not clear, several researches have indicated that capsaicin has a therapeutic and preventive effect on cancer. Several in vivo studies have used rodent samples and supported the antitumor activity of capsaicin [7]. The proposed anticancer mechanisms of capsaicin include increased inhibition of the cell division and activation of apoptosis, but the main mechanism is still not fully understood [8].

## Materials and methods

In this study, we induced squamous cell carcinoma in the buccal pouch of 40 golden Syrian hamsters. And investigate the expression of Ki-67 and Bcl-2 proteins on samples with the administration of chili extract (capsaicin) with the degree of its expression without capsaicin in hamsters during the development of oral cancer in them by 7,12-dimethylbenz(a)anthracene (DMBA). The sample consisted of 40 hamsters divided into two groups. In the first group (the control) with 20 hamsters, oral cancer was induced by DMBA without applying capsaicin. In the second group (study group) with 20 hamsters, cancer was induced by DMBA and then applying capsaicin in the digestive tract. Experimental animals were sacrificed in groups of five hamsters and at intervals (five after two weeks, five after six weeks, five after 10 weeks, and five after 14 weeks).

Chili extract (capsaicin)

The active ingredient was extracted from red pepper in the pathological laboratory in Faculty of Dentistry, Damascus University, depending on the solubility of capsaicin in alcohol. Using a syringe, the extract was left for six weeks covered with blotting paper (to protect against impurities) until all the alcohol had evaporated, thus we obtained a pure extract of hot pepper [9]. Capsaicin was administered into the digestive tract directly by a 1 cm^3^ syringe after diluting the extract with vegetable oil (sunflower oil) according to the recommended dose to hamsters to avoid local and toxic effects of increasing the dose [10]. We diluted 1 ml of concentrated capsaicin in 100 ml of sunflower oil as we found this dose to be the preferred dose for the experimental animals and no adverse reaction occurred in them. After sacrificing the hamster, the right buccal pouch was excised and prepared to be studied histologically.

Histological slides

Control Sample

The slides of the control sample stained with Ki-67 and Bcl-2 were examined by light microscopy and it was found that most of these cells show nuclear expression of the stain Ki-67 and both nuclear and cytoplasmic expression of stain Bcl-2.

Samples from the study group

Slides stained with Ki-67 and Bcl-2 were examined by light microscopy according to the three pre-divided groups based on hematoxylin-eosin staining. Five histological squares of each report were studied on the x40 lens. The number of cells that showed expression in the tissue layers of oral dysplasia was manually counted, and after the cell count was completed for the five studied squares of each preparation, the resulting numbers were placed within the appropriate field in tables designed on Microsoft Excel. It was considered that the percentage of cells in each report is the average percentage in the five studied squares.

## Results

Control sample

**Figure 1:**
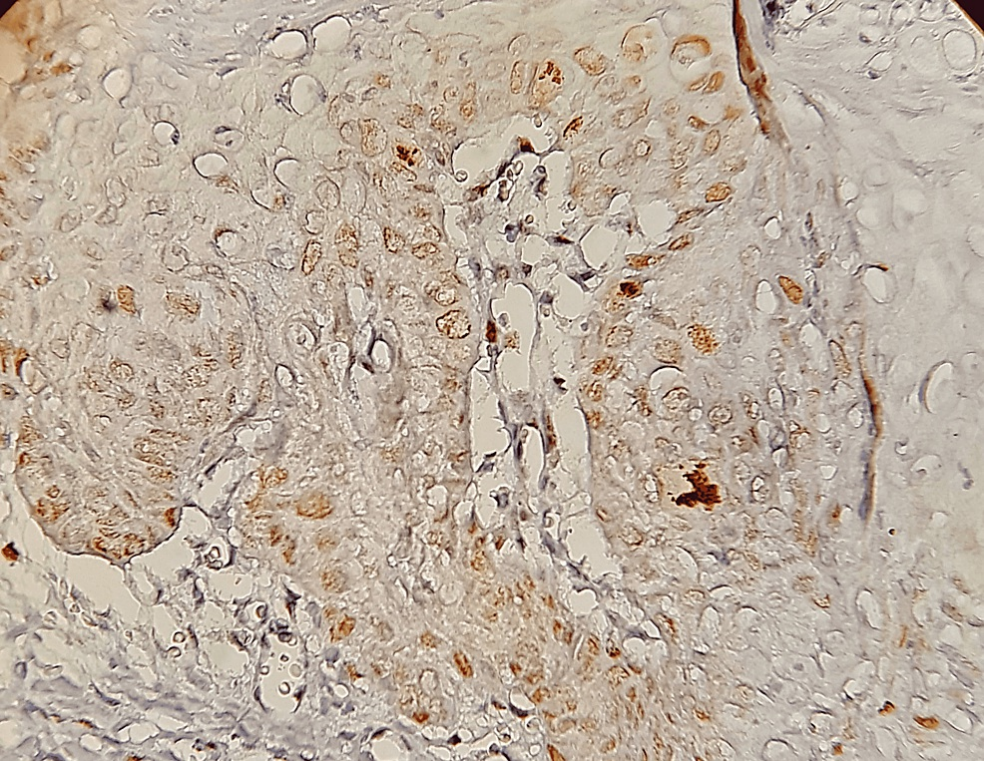
The expression of Ki-67 in the control group after 10 weeks.

**Figure 2:**
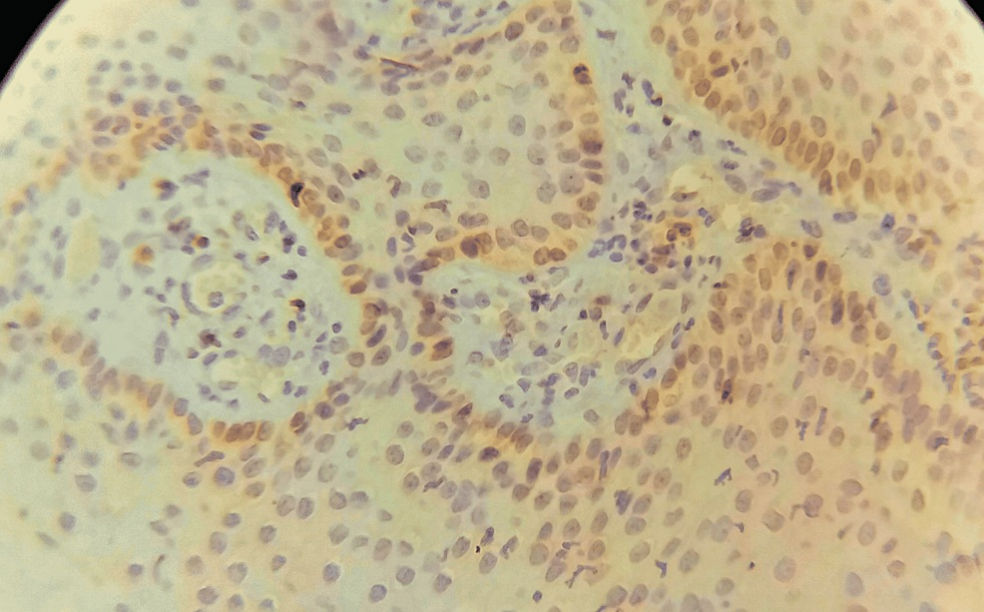
The expression of Bcl-2 in the control group after 10 weeks.

All samples of the control group showed positive expression of Ki-67 and Bcl-2 stain, and the expression was distributed in basal and suprabasal cells in the two-week samples, basal and suprabasal and some squamous cells in the six-week samples and extended to the granular layers in the 10-week samples, while the expression included the entire layers of the epidermis in the 14 samples a week (Figures 1, 2).

Study sample

**Figure 3:**
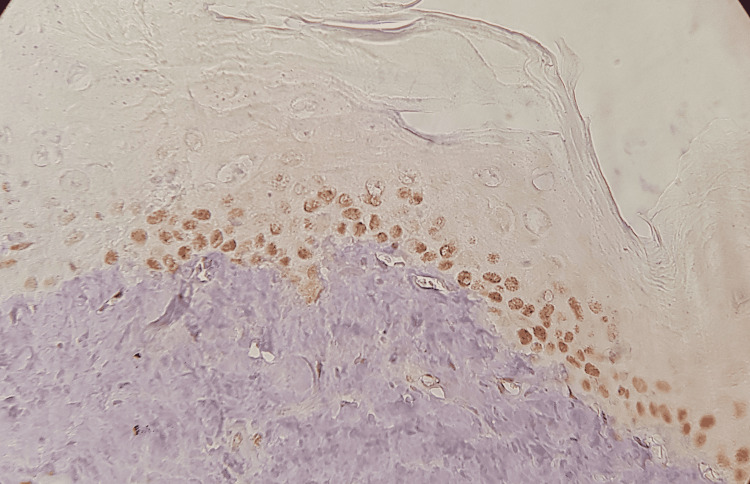
The expression of Ki-67 in the study group after 10 weeks.

**Figure 4:**
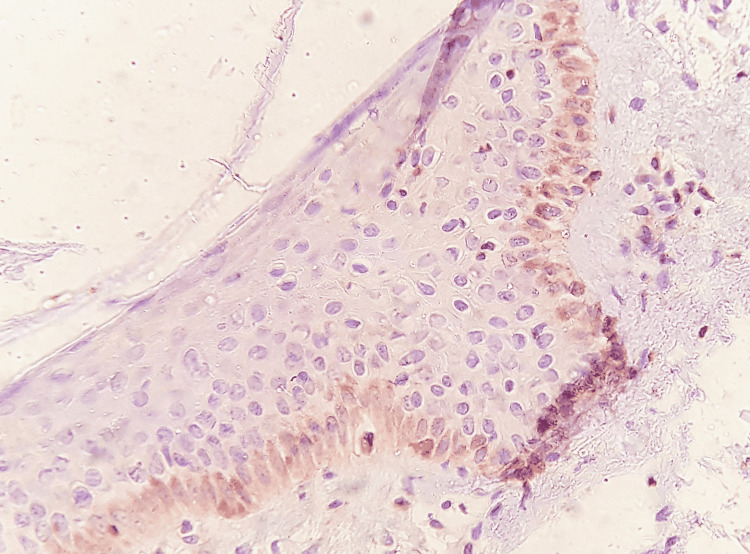
The expression of Bcl-2 in the study group after 10 weeks.

The expression was distributed in the basal layers in the two-week samples and the basal and suprabasal layers in the six-week samples, and the expression reached the spinous layers in the 10-week samples, while the expressiveness included the entire layers of the epidermis in the 14-week samples and no islands appeared pre-cancerous or expressive palpable in connective tissue (Figures 3, 4).

A one-way (ANOVA) test was conducted to study the significance of the differences in the mean values ​​of Ki-67 expression between groups for the studied time period (after two weeks, after six weeks, after 10 weeks, and after 14 weeks) in the study sample (Table 1).

**Table 1:** The studied variable = Ki-67 protein expression ratio. The results of the one-sided analysis of variance (ANOVA) test study the significance of the differences in the average values of Ki-67 expression between groups of the studied time period (after two weeks, after six weeks, after 10 weeks, and after 14 weeks) in the study sample of the preventive effect capsaicin, according to the studied group.

The studied group	The time period studied	The number of tissue biopsies	Average calculation	Standard deviation	Minimum	The highest rate	Computed f-value	p-Value	The significance of the differences
Application of capsaicin with the carcinogen	Two weeks later	5	10.6	3.65	5	15	13.910	0.000	There are significant differences
	Six weeks later	5	14.20	3.19	10	18			
	Ten weeks later	5	16.60	2.88	13	20			
	Fourteen weeks later	5	32.40	10.06	25	50			
Carcinogen application only (control group)	Two weeks later	5	20.40	2.70	18	25	9.973	0.001	There are significant differences
	Six weeks later	5	51.00	21.04	25	75			
	Ten weeks later	5	67.80	12.91	45	75			
	Fourteen weeks later	5	37.40	14.01	25	55			

As it is noted in Table 1, the p-value is smaller than the value of 0.05 regardless of the group studied, i.e., at the 95% confidence level, there are statistically significant differences in the average values ​​of Ki-67 protein expression ratio between at least two of the four studied time period groups (after two weeks, after six weeks, after 10 weeks, and after 14 weeks), whatever group was studied in the study sample.

A one-way (ANOVA) was conducted to study the significance of the differences in the mean values ​​of Bcl-2 expression between groups of the studied time period (after two weeks, after six weeks, after 10 weeks, and after 14 weeks) in the study sample (Table 2).

**Table 2:** The studied variable = Bcl-2 protein expression ratio. The results of the one-sided analysis of variance (ANOVA) test study the significance of the differences in the average values of Bcl-2 protein expression between groups of the studied time period (after two weeks, after six weeks, after 10 weeks, and after 14 weeks) in the study sample of the preventive effect capsaicin, according to the studied group.

The studied group	The time period studied	The number of tissue biopsies	Average calculation	Standard deviation	Minimum	The highest rate	Computed f-value	p-Value	The significance of the differences
Application of capsaicin with the carcinogen	Two weeks later	5	3.60	8.05	0	18	10.374	0.000	There are significant differences
	Six weeks later	5	3.00	6.71	0	15			
	Ten weeks later	5	9.00	12.45	0	25			
	Fourteen weeks later	5	28.20	2.05	25	30			
Carcinogen application only (control group)	Two weeks later	5	32.60	3.29	30	38	81.934	0.000	There are significant differences
	Six weeks later	5	47.60	5.59	40	55			
	Ten weeks later	5	70.60	4.39	65	75			
	Fourteen weeks later	5	72.80	5.40	65	79			

It is noted in the above table that the p-value is much smaller than the value of 0.05 regardless of the group studied, i.e., at the 95% confidence level, there are statistically significant differences in the average values ​​of Bcl-2 protein expression between at least two of the four studied groups of time period (after two weeks, after six weeks, after 10 weeks, and after 14 weeks), whatever group was studied in the study sample.

## Discussion

The oral mucosa consists of stratified epithelium; this stratification is the result of successive cell proliferation and differentiation [11]. Proliferation is characteristic of generating cells in the basal layer of the stratum corneum. Differentiation begins when dividing cells detach from the extracellular matrix, and when dividing cells mature, they are pushed toward the outer surface by pressure generated by cell proliferation in the basal layer [12]. Cell proliferation, a vital biological process is one of the important factors that aid in the histological classification of tumors and is a potential indicator of tumor response to treatment and relapse. Several studies have reported that abnormal cell proliferation may be a predictor of tumorigenesis [13]. Several immunomarkers are used to detect cellular proliferation. Ki-67 is considered one of the most available and reliable in the study of cellular proliferation [14]. On the other hand, apoptosis is an essential barrier to cancer development and progression. Many types of cancer suppress cellular death signals and enhance non-death signals, making cancer cells resistant to apoptosis [15]. The core mitochondrial death pathway and the extrinsic death receptor pathway are the two major signaling systems that activate apoptosis. In particular, the mitochondrial pathway is involved in the complete implementation of cell death, so the mitochondria have been called the "gatekeeper" of apoptosis [16]. The mechanism of apoptosis, related proteins, and signaling pathways for mitochondrial death have become promising targets for new therapeutic procedures related to cancer. If, by means of chemicals, we can target factors that contribute to suppressing the proliferation of cancer cells, and inducing apoptosis in those cells, we may be able to stop tumor growth and even eliminate cancer cells. Phytochemicals are widely available in nature and are cheaper and less harmful to healthy cells than other chemicals manufactured in laboratories that are used in chemotherapy programs for cancer patients, which exhausts the patient's body and fatigues his psyche. Capsaicin is a naturally occurring substance (hot red pepper), which has been proven in many studies its anti-cancer effect by targeting the signaling pathways of cancer cells and eliminating them without harming healthy cells. In our study, we found that the application of capsaicin into the hamster's diet significantly suppressed the proliferation of squamous cell carcinoma (SCC) cells. By comparing the degrees of dysplasia that appeared in the different stages of oral squamous cell carcinoma (OSCC) development, we found that the degree of expression of Ki-67 in the buccal pouch of the hamster that took capsaicin was lower than those in control group, and we found statistically significant differences between its expression in the control group and the study group in all the time periods in which experimental animals were sacrificed. Our study is in agreement with that of Chen et al., where capsaicin showed clear efficacy in inhibiting cancer cell division and decreasing Ki-67 expression in breast cancer [17]. Our study also agreed with that of Caetano et al., where capsaicin showed an inhibitory effect on the proliferation of colon cancer cells induced in rats [18]. We found that capsaicin induced apoptosis in cancer cells by suppressing the expression of the mutant Bcl-2 gene. Which led to a significant decline in the progression of cancer clinically and histologically. The results of our study are in agreement with that of Kamaruddin et al., who found an antitumor therapeutic role for oral squamous cell carcinoma by suppressing cell proliferation and activating apoptosis [19].

Research limitations

Our study investigated the effect of capsaicin on the rate of proliferation and apoptosis of oral squamous cell carcinoma cells in hamsters, which is very similar to oral squamous cell carcinoma in humans, but the metabolic activity may differ between human and hamster cells. Also, the incidence of cancer caused by DMBA is faster than the carcinogens that can lead to cancer in humans.

## Conclusions

In this study, we found by comparing the two study groups that adding chili extract (capsaicin) to the diet of Syrian hamsters activates apoptosis by decreasing the expression of Bcl-2 protein and inhibits the proliferation of cancer cells by decreasing the expression of Ki-67 protein in all stages of oral squamous cell carcinoma development. We also found that the degree of cancer malignancy differed significantly between the two study groups, i.e., the addition of capsaicin reduces the malignancy of cancer at all stages.

## References

[1] Tantamango-Bartley Y, Jaceldo-Siegl K, Fan J, Fraser G (2013). Vegetarian diets and the incidence of cancer in a low-risk population. Cancer Epidemiol Biomarkers Prev.

[2] van't Veer P, Jansen MC, Klerk M, Kok FJ (2000). Fruits and vegetables in the prevention of cancer and cardiovascular disease. Public Health Nutr.

[3] Clarke JD, Dashwood RH, Ho E (2008). Multi-targeted prevention of cancer by sulforaphane. Cancer Lett.

[4] Aggarwal BB, Shishodia S (2006). Molecular targets of dietary agents for prevention and therapy of cancer. Biochem Pharmacol.

[5] Reyes-Escogido Mde L, Gonzalez-Mondragon EG, Vazquez-Tzompantzi E (2011). Chemical and pharmacological aspects of capsaicin. Molecules.

[6] Opheim MN, Rankin JW (2012). Effect of capsaicin supplementation on repeated sprinting performance. J Strength Cond Res.

[7] Kim CS, Kawada T, Kim BS, Han IS, Choe SY, Kurata T, Yu R (2003). Capsaicin exhibits anti-inflammatory property by inhibiting IkB-a degradation in LPS-stimulated peritoneal macrophages. Cell Signal.

[8] Clark R, Lee SH (2016). Anticancer properties of capsaicin against human cancer. Anticancer Res.

[9] Lau BB, Panchompoo J, Aldous L (2015). Extraction and electrochemical detection of capsaicin and ascorbic acid from fresh chilli using ionic liquids. New J Chem.

[10] Zhang Z, Huynh H, Teel R (1997). Effects of orally administered capsaicin, the principal component of capsicum fruits, on the in vitro metabolism of the tobacco-specific nitrosamine NNK in hamster lung and liver microsomes. Anticancer Res.

[11] Shang L, Deng D, Buskermolen JK (2018). Multi-species oral biofilm promotes reconstructed human gingiva epithelial barrier function. Sci Rep.

[12] Byrd KM, Piehl NC, Patel JH (2019). Heterogeneity within stratified epithelial stem cell populations maintains the oral mucosa in response to physiological stress. Cell Stem Cell.

[13] Feng Y, Spezia M, Huang S (2018). Breast cancer development and progression: Risk factors, cancer stem cells, signaling pathways, genomics, and molecular pathogenesis. Genes Dis.

[14] Thakur B, Kishore S, Dutta K, Kaushik S, Bhardwaj A (2017). Role of p53 and Ki-67 immunomarkers in carcinoma of urinary bladder. Indian J Pathol Microbiol.

[15] Bock FJ, Tait SW (2020). Mitochondria as multifaceted regulators of cell death. Nat Rev Mol Cell Biol.

[16] Zhou X, Jiang W, Liu Z, Liu S, Liang X (2017). Virus infection and death receptor-mediated apoptosis. Viruses.

[17] Chen M, Xiao C, Jiang W (2021). Capsaicin inhibits proliferation and induces apoptosis in breast cancer by down-regulating FBI-1-mediated NF-κB pathway. Drug Des Devel Ther.

[18] Caetano BF, Tablas MB, Pereira NE, de Moura NA, Carvalho RF, Rodrigues MA, Barbisan LF (2018). Capsaicin reduces genotoxicity, colonic cell proliferation and preneoplastic lesions induced by 1,2-dimethylhydrazine in rats. Toxicol Appl Pharmacol.

[19] Kamaruddin MF, Hossain MZ, Mohamed Alabsi A, Mohd Bakri M (2019). The antiproliferative and apoptotic effects of capsaicin on an oral squamous cancer cell line of Asian origin, ORL-48. Medicina (Kaunas).

